# Preparation and Evaluation of Physical and Chemical Properties of Resin Plugging System Suitable for Formation Plugging of Malignant Lost Circulation

**DOI:** 10.3390/gels10100633

**Published:** 2024-09-30

**Authors:** Wei Gao, Mo Wang, Shixin Lian, Yingrui Bai, Jingbin Yang

**Affiliations:** 1 Key Laboratory of Enhanced Oil Recovery in Carbonate Fractured-Vuggy Reservoirs, Sinopec, Urumqi 830011, China; 2 Sinopec Northwest Company of China Petroleum and Chemical Corporation, Urumqi 830011, China; 3 School of Petroleum Engineering, China University of Petroleum (East China), Qingdao 266580, China

**Keywords:** lost circulation, resin, mechanical property, curing effect, temperature resistance

## Abstract

Lost circulation is one of the important problems that restricts the speed and efficiency of oil and gas drilling and production. In this study, a resin plugging system was successfully developed for lost circulation formation. The resin plugging system showed excellent performance under high temperature and pressure conditions. The experimental results showed that the compressive strength of the resin plugging material can reach 9.23 MPa after curing, which is significantly higher than that of the traditional polymer gel material. The resin material can achieve effective curing in the temperature range of 60 °C to 100 °C, and the curing time decreases with the increase of temperature and only needs 3.46 h at 140 °C. The microstructure results showed that the resin material can form a chain or three-dimensional network structure after curing, which can effectively increase the toughness and strength of the cured plugging layer. Infrared and thermogravimetric analysis further confirmed the thermal stability of the chemical bonds in the material, and the initial decomposition temperature was about 241 °C, indicating that it had good thermal stability at about 300 °C. In addition, the effects of curing temperature, salinity, and drilling fluid pollution on the properties of the resin plugging agent were also investigated. The results showed that curing agent dosage and curing temperature are the key factors affecting curing time, while salinity and drilling fluid pollution affect the curing strength and overall properties of the materials. After adding 20% KCl polysulfonate drilling fluid, the compressive strength of the consolidated body decreased to 4.55 MPa. This study can provide an efficient and reliable plugging solution for malignant loss formation.

## 1. Introduction

The problematic flow of drilling fluid, also known as lost circulation, is a major challenge during oil and gas drilling, particularly when drilling oil and gas reservoirs with fracture characteristics [1]. Lost circulation and its associated problems account for a significant portion of the overall drilling costs [2]. According to relevant studies, ≈25% of wellheads in drilling operations in North America experience severe lost circulation. In a fractured carbonate formation in the Middle East, the percentage is as high as 40%, and in the Tarim Basin of China, the percentage of lost circulation is even more than 60% [3]. Lost circulation not only causes significant economic losses but also significantly increases the time spent on drilling operations. If the lost circulation problem is not dealt with in a timely and effective manner, it may trigger a pressure imbalance within the wellbore. The imbalance in turn induces serious well-control incidents such as borehole collapse, well surging, or even blowout [4,5]. The lost time owing to lost-circulation events accounts for more than 70% of the total lost time owing to all accidents during the drilling process. The mean direct economic loss in 2017 and 2018 exceeded USD 4 billion/year, which further highlights the urgency for a solution to the lost-circulation problem [6,7]. Lost circulation has become a global problem restricting oil exploitation, which affects the benefit and progress of oil and gas exploitation to a great extent.

Polymer gel plugging materials are becoming the most popular materials because of their flexible adjustability, easy pumping, and adaptive crack size and shape [8,9,10]. Polymer gels are generally composed of polymerization main agent, crosslinking agent, initiator, and a variety of materials, and can achieve different characteristics through the introduction of specific components [11]. Under normal circumstances, polymer gel plugging agents need to have certain properties such as temperature resistance and salt resistance according to the different geological conditions [12]. Dai et al. prepared dispersed particle gel (DPG) and studied its macroscopic plugging performance [13]. It was found that when the Young’s modulus of the DPG particles increased from 82 Pa to 328 Pa, the plugging rate increased from 91.46% to 97.10% [14]. However, when the Young’s modulus of the DPG particles exceeded 328 Pa, with the increase of the Young’s modulus of DPG particles, the further improvement of the plugging rate was not significant. Zhu et al. took preformed degradable gel particles (DPPGs) as the research object and studied their salt expansion resistance indoors [15]. They concluded that DPPGs showed good swelling capacity in a wide brine salinity range (20,000–400,000 ppm) and could be completely degraded, and the degradation time extended with the increase of temperature [16]. By studying the matching relationship between adaptive microgel (SMG) and fractured core, Chen et al. divided plugging into direct plugging, bridge plugging, superimposed plugging, and hydrodynamic plugging according to the amount of SMG and relative pore throat size [17]. Esfahlan et al. classified different types of PPG, discussed and evaluated the main parameters of gel properties, including particle size, swelling capacity, rheological properties and plugging efficiency, and concluded that PPG could be combined with low salinity water flooding, surfactant flooding, weak polymer gel flooding, polymer flooding, and other oil flooding methods [18].

Thermoset resin materials show great application prospects in the field of oil drilling operations [19,20]. This is due to their inherent versatility, excellent mechanical properties, heat resistance, and significantly improved chemical resistance after modification. They can also be applied under different molding temperatures and pressure conditions [21,22,23]. Li et al. synthesized a styrene–butadiene resin/nano-SiO_2_ composite as a plugging agent for oil-based drilling fluid using the continuous emulsion polymerization method. The composite effectively enhanced plugging efficiency in shale formations [24]. The resin plugging agent can enter into the shale’s nanopores and significantly reduce fluid invasion, thus enhancing wellbore stability. Huang et al. synthesized an acrylic resin/SiO_2_ nanocomposite with a core–shell structure in water-based drilling fluid [25]. This improved the efficiency of pore plugging during shale gas drilling, reduced fluid intrusion, and further enhanced the stability of the wellbore [26]. Liu et al., on the other hand, used in situ polymerization technology to modify epoxy resin to prepare nano-SiO_2_-reinforced cement injection materials. This shortened the setting time of the composite slurry and improved its stability, in addition to slowing down the hydration process of the cement and enhancing the early compressive strength of the composite slurry [27]. Batista et al. used polyethylene terephthalate-modified polyester resins as plugging materials, which exhibited high compressive strengths and low viscosities [28]. They provided an innovative method for erosion control as well as for abandonment and remediation applications. Lv et al. developed an underwater high-temperature and slow-consolidating epoxy-resin plugging system. The system can be easily cured in an environment that simulates formation fractures. The epoxy resin consolidated at 120 °C has good compressive strength and effectively plugs fractures, making it suitable for drilling-fluid plugging in the oil and gas drilling process [29]. Knudsen et al. developed a thermosetting resin plugging agent to effectively treat heavy-oil mud loss in offshore gas wells in the Middle East [30]. This demonstrates the practicality and potential of thermosetting resin materials for solving the problem of drilling fluid loss.

In this study, a resin plugging system for lost circulation was developed to solve the existing problem of drilling fluid loss [31,32]. The resin plugging system was designed to overcome the limitations of conventional materials and provide a fast, robust and durable seal in the harsh conditions of deep wells [33,34]. This study focuses on constructing and validating a resin based plugging system that performs better than conventional materials. This includes evaluating the curing behavior, mechanical properties, and chemical stability of resin plugging systems under simulated downhole conditions, as well as assessing their effectiveness in treating severe lost circulation.

## 2. Results and Discussion

### 2.1. Preparation of Resin Plugging System

#### 2.1.1. Resin Matrix Optimization

The curing performance of different thermosetting resins was investigated. Five kinds of thermosetting resins, namely, urea–formaldehyde resin, epoxy resin, phenolic resin, polyvinyl alcohol (PVA) resin, and melamine resin, were selected and cured at 60, 80, and 100 °C by adding a certain amount of suitable curing agent. The curing results are shown in Table 1. It can be seen that none of the five resins can be cured at 40 °C. As the temperature increased, the urea–formaldehyde resin, phenolic resin, PVA, and epoxy resin could be cured, but only the urea–formaldehyde and phenolic resins maintained high strength. At 100 °C, several other resins were cured (but with low strength) or were unable to be cured.

**Figure 1:**
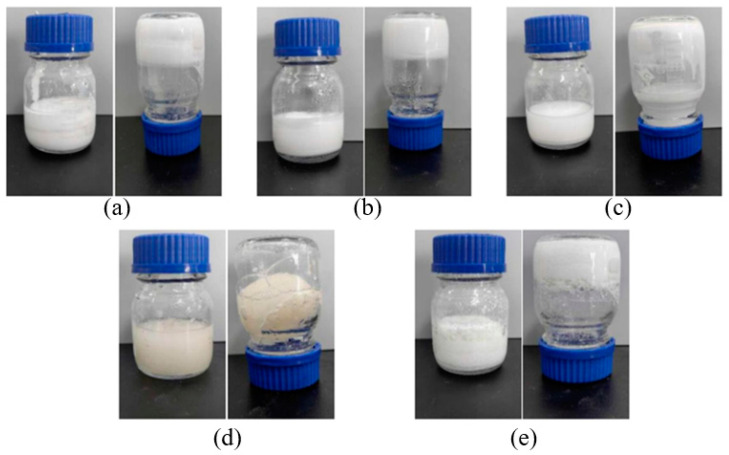
Results of static curing at 60 °C for five resins. (**a**) urea resin, (**b**) phenolic resin, (**c**) melamine resin, (**d**) polyvinyl resin, (**e**) epoxy resin.

As shown in Figure 1, the urea–formaldehyde and phenolic resins are better cured at 60 °C than the other resins.

**Figure 2:**
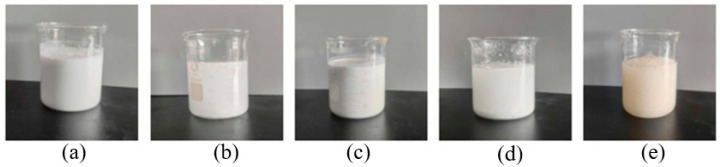
Stratification of five resins after 8 h of resting. (**a**) urea resin, (**b**) phenolic resin, (**c**) epoxy resin, (**d**) polyvinyl resin, (**e**) melamine resin.

The resin plugging system needs to be well stabilized and dispersed before it enters the destination layer to ensure that all components remain in the curable concentration range. Thus, the settling stability of the five resins was studied. The five resins were dispersed in 20% aqueous solution (by mass), and the dispersions were monitored for varying durations of standing. The results are shown in Table 2. At 1 h of standing, the urea–formaldehyde, phenolic, and epoxy resins were relatively well dispersed, while the PVA resin appeared to be partially precipitated, and the melamine resin was cured. When the dispersion time was 8 h, only the urea–formaldehyde resin and epoxy resin were dispersed uniformly, and a small amount of phenol–formaldehyde resin also began to precipitate. The resin after standing for 8 h is shown in Figure 2.

According to the above results, the melamine resin could not be cured, settlement stability was poor, and it was easily stratified. PVA resin cured too fast and had a strength of five. Neither of them could meet the requirements, so these two resin substrates were excluded. Three materials, urea–formaldehyde resin, epoxy resin, and phenolic resin, were used in subsequent experiments. Different kinds of resin matrix were added to the water, stirring at 600 rpm for 5–15 min to disperse evenly. Then, thiourea, lithium bentonite, curing agent, crosslinking agent, nano silica, walnut shell and quartz sand were added. Triethanolamine curing agent was selected for the epoxy resin. Phenolic resin used sodium hydroxide curing agent. Urea–formaldehyde resin used a latent curing agent composed of sulfonic acid and ammonium curing agent. Stirring was continued for 5–15 min until even dispersion, and then poured into the cylindrical high-temperature mold, put into the variable frequency high-temperature roller heating furnace, rolled and heated at 100–140 °C for a period of time, taken out and the curing observed. The specific curing conditions are shown in Table 3.

From the perspective of curing conditions, under the conditions of 100–140 °C, the epoxy resin and phenolic resin failed to meet the molding requirements and the strength requirements required for pressure plugging. However, urea–formaldehyde resin cured well and had a certain strength, and combined with its good sedimentation stability, it was finally selected as the resin matrix for this experiment.

The curing of urea–formaldehyde resin with different concentrations was further studied. The mass fraction of the resin was 12%, 15%, 20%, and 25% by adding different masses of urea–formaldehyde resin matrix into water. Stirring at 600 RPM for 5–15 min to disperse evenly was carried out. Then, thiourea, lithium bentonite, latent curing agent, crosslinking agent, nano silica, walnut shell, and quartz sand were added, stirring was continued for 5–15 min until evenly dispersed. The mass was poured into the cylindrical high-temperature mold, respectively, into the variable frequency high temperature roller heating furnace at 100 °C with rolling heating for a period of time, and then taken out to observe the curing. The curing condition is shown in Table 4.

The experimental results show that the curing strength of the resin plugging system increases with the increase of resin concentration. However, when the resin concentration increased to 25%, the curing strength increased very little. Considering the economic cost and other factors, 20% was selected as the best resin concentration in this experiment.

#### 2.1.2. Curing Agent Selection

The curing agent has a huge impact on the curing effect of the resin system, so the first step was to optimize the type of curing agent. No curing agent, ammonium chloride, a mixture containing an ammonium compound (diammonium hydrogen phosphate mixed with ammonium persulfate in different ratios), and a latent curing agent (sulfonic acid and ammonium curing agent compounded together) were studied. The four plans are shown in Table 5.

**Figure 3:**
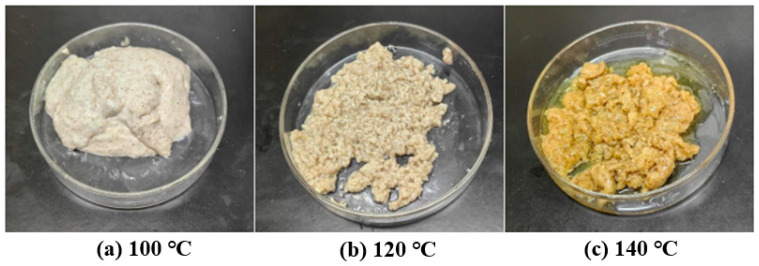
Curing of resins at different temperatures without curing agent.

The curing of the resin plugging agent was investigated at 100, 120, and 140 °C without adding any curing agent. The results show that curing was unsuccessful at 100–140 °C without the addition of a curing agent, as shown in Figure 3.

**Figure 4:**
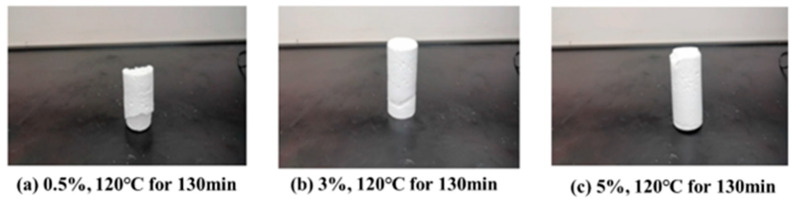
Curing of different concentrations of ammonium chloride curing agent.

The curing effect of different concentrations of ammonium curing agent on resin plugging agent was studied. In the experiment, ammonium chloride with 0.5%, 3%, and 5% mass fraction was selected as the curing agent for urea–formaldehyde resin, and the curing effect was studied at 120 °C for 130 min. The experimental results are shown in Figure 4.

The results showed that with the addition of 0.5% ammonium chloride as a curing agent, the solidus fractured and was relatively weak. With the addition of 3% and 5% curing agent, the solidus cured well and was stronger.

**Figure 5:**
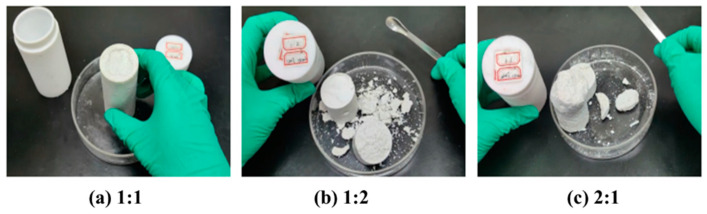
Curing of resin plugging agent using mixed curing agents containing ammonium persulfate + diammonium hydrogen phosphate in varying ratios.

The curing effect of different ratios of complex ammonium curing agent (ammonium persulfate and diammonium hydrogen phosphate) on resin plugging agent was studied. The ratios of ammonium persulfate and diammonium hydrogen phosphate were 1:1, 1:2, and 2:1, respectively. The curing effect was studied by hot rolling at 120 °C for 130 min, respectively, as shown in Figure 5.

The results of the study show that mixing ammonium persulfate and diammonium hydrogen phosphate in a 1:1 ratio formed an intact solid with high strength, whereas solids were formed when mixed in 1:2 and 2:1 proportions. However, the solids were more brittle, and all of them underwent fracture breakage to varying degrees. During the reaction, the addition of ammonium persulfate can initiate the curing of the urea–formaldehyde resin. This is due to the chemical reaction between the hydroxymethyl or methylene groups in the resin and the reactive groups in the ammonium persulfate, forming a cross-linked structure. This curing process involves the formation of chemical bonds, usually covalent bonds. Ammonium persulfate acts as a catalyst in this process and accelerates the curing reaction because it is a salt system composed of a strong acid and weak base. In addition, ammonium persulfate has redox properties, which can initiate a variety of complex chemical reactions that contribute to the generation of more stable cross-linked structures.

**Figure 6:**
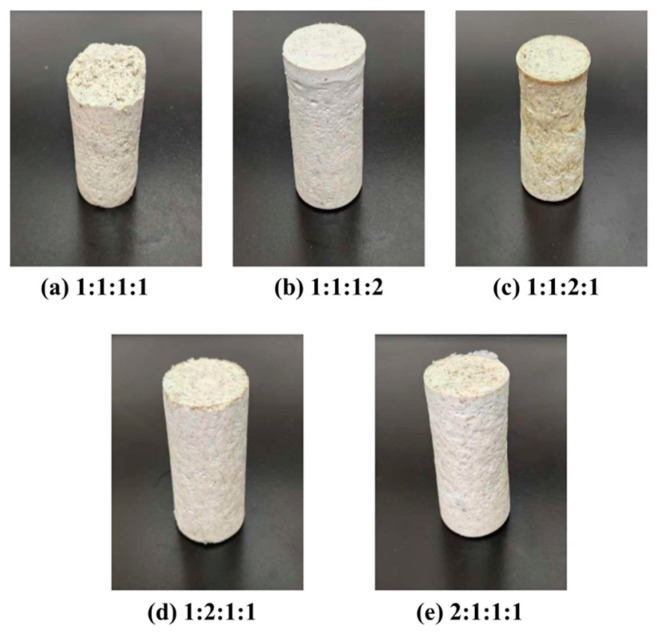
Curing of latent curing agents with varying ratios.

The curing effect of varying ratios of latent curing agents on high-temperature and high-strength resin plugging agents was investigated. Five ratios of *p*-toluenesulfonic acid:hexamethylenetetramine:diethanolamine:ammonium persulfate in the latent curing agent were selected for the experiments: 1:1:1:1, 1:1:1:2, 1:2:2:1, 1:2:1:1, and 2:1:1:1. The curing effect was investigated after heat-rolling for 130 min at 120 °C. The curing results are shown in Figure 6.

The results of the study show that the strongest resin cement formed when the ratio of latent curing agents (including *p*-toluenesulfonic acid, hexamethylenetetramine, diethanolamine, and ammonium persulfate) was 1:1:1:2. By contrast, when the ratios were 1:1:1:1, 1:2:1:1, and 2:1:1:1, the strength of the solid was relatively low, although the solid could also be formed. This is due to the low content of *p*-toluenesulfonic acid and relatively high content of ammonium persulfate in the ratio of 1:1:1:2, which makes the latent curing agent remain relatively stable at room temperature and unable to immediately react with the urea–formaldehyde resin. However, under external stimulation (e.g., heating, light), it reacts with hydroxymethyl or methylene groups in the urea–formaldehyde resin to form a crosslinked structure, thus being cured. The cured urea–formaldehyde resin has a high strength and hardness that can meet the specific requirements.

The strength of four kinds of curing agents involved in the reaction to form the consolidated body was further studied and compared. Since Plan 1 (without curing agent) failed to form a complete solid, only the last three plans were selected to determine the maximum compressive strength. The compressive strength test results are shown in Table 6.

The results show that the selected latent curing agent has the best compressive strength (up to 7.13 MPa). Therefore, the latent curing agent was chosen for subsequent experiments.

#### 2.1.3. Crosslinking Agent Selection

The effect of cross-linking agent on the curing of high-temperature, high-strength curable resin plugging agent was investigated to optimize the appropriate cross-linking agent ratio. In this experiment, two liquid cross-linking agents, hydroxyethyl methacrylate and divinylbenzene, were used. One, or both of them, was added to each experimental plan, and their curing effects were studied after curing at 120 °C for 180 min. The results of the study are shown in Table 7.

The results revealed that when either hydroxyethyl methacrylate or divinylbenzene is added, either a solid does not form, or a solid with low strength and breakage is formed, respectively. Therefore, neither can meet the demands of lost circulation plugging. When the two cross-linking agents are added simultaneously, a complete solid with a compressive strength of up to 7.07 MPa is formed, demonstrating a better plugging effect. Therefore, Plan 3 (0.5% hydroxyethyl methacrylate + 0.2% divinylbenzene as the cross-linking agent) was adopted in subsequent experiments.

#### 2.1.4. Optimization of Flow-Regulator Concentration

As the resin plugging agent is cured in the fracture space, it should not be easily diluted. The resin’s viscosity could be increased before curing, but it needs to have a relatively good flow effect in the surface pipeline and the wellbore. Therefore, shear thinning/static thickening of the flow regulator was adopted to control the viscosity. The experimental effect of the flow conditioner and its concentration on the shear thixotropy of the resin plugging agent was evaluated by investigating the variation in the viscosity of the resin plugging agent with shear rate.

**Figure 7:**
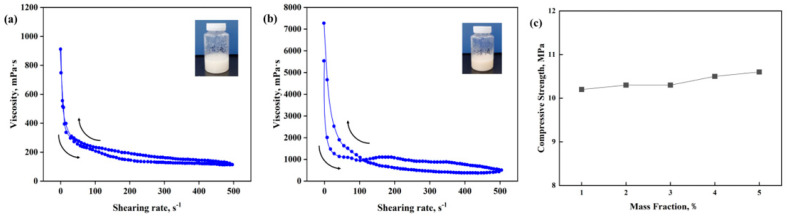
Concentration selection of flow-conditioning agent. (**a**,**b**) Thixotropy of the resin plugging agent system with 1% and 3% flow conditioner added. (**c**) Effect of flow conditioner concentration on curing strength.

The research results show that the flow regulator will impart shear thinning/static thickening thixotropic properties to the high-temperature-resistant, high-strength resin plugging agent. The impact on the curing strength is relatively small. The initial viscosity of the resin plugging agent under 1% flow regulator was 950 mPa s, and the viscosity of the resin plugging agent decreased to ≈160 mPa s when the shear rate was increased to 500 s^−1^. The initial viscosity of the resin plugging agent with 3% flow regulator added was 5700 mPa s, and the viscosity of the resin plugging agent decreased to ≈600 mPa s when the shear rate was increased to 500 s^−1^. The viscosity of the resin plugging agent recovered well with the decrease in shear rate; however, the initial viscosity of the resin plugging agent was too high when 3% flow regulator was added, making the resin difficult to pump. At the same time, the experimental results also showed that the resin plugging agent can be cured well under different flow type regulator concentrations, and the curing strength is above 10 MPa. Therefore, the rheology modifier concentration selected for the resin system was 1%. The experimental results are shown in Figure 7.

#### 2.1.5. Selection of Solid Filler Materials

To apply practically a resin plugging agent in order to reduce the cost of controllable curing resin and increase the curing strength of controllable curing resin, a certain amount of solid filler material is usually added. In this study, filler materials such as quartz sand, barite, walnut shells, nano-silica, and fibers were selected for their stable dispersion in the resin solution under the action of rheology modifying agents, which can increase the curing strength of the resin. The filler materials selected for the experiments are listed in Table 8.

**Figure 8:**
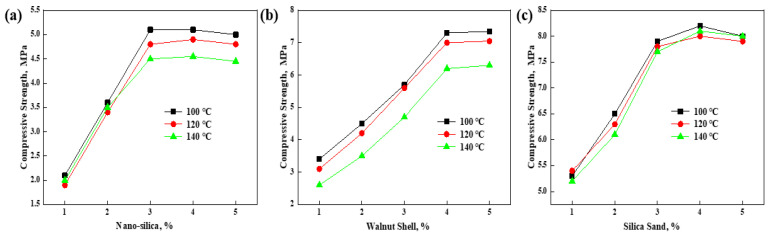
Effects of (**a**) nanosilica, (**b**) walnut shell, and (**c**) quartz sand on cured strength.

In this study, nanosilica, walnut shells, and quartz sand were combined and used as the solid filler material of the resin plugging agent system. First, heat-roll curing experiments were carried out at 100, 120, and 140 °C. The filler material ratio was selected based on the curing strength of the three material concentrations, as shown in Figure 8. The experimental results showed that when the concentration of nano-silica is 3%, the concentration of walnut shell is 4%, and the concentration of quartz sand is 3%, the compressive strength of the resin plugging system after curing has reached a peak. Therefore, the type and concentration of the filling material was determined as follows: 3% nano-silica +4% walnut shell +3% quartz sand. The density of the resin consolidation and plugging system formed by adding filler material was 1.11 g/cm^3^.

Based on the five material selection experiments for curing high-temperature-resistant, high-strength curable resin plugging agent, the best curing agent was determined to have the following composition: 20% resin + 1% rheology modifier + 3% latent curing agent + 0.7% crosslinking agent + 10% solid filler material. An electronic balance was used to weigh the resin matrix of the desired quality, poured into a glass beaker containing deionized water, and mixed with a blender for 5–10 min. Afterward, the flow-regulating agent, latent curing agent, cross-linking agent, nano-silicon dioxide, walnut shells, and quartz sand were added in turn and mixed for 5–15 min until the material was completely and uniformly dispersed, producing the lost-circulation-material fluid samples. A mold-releasing agent (urea–formaldehyde resin special mold-release agent or dimethyl silicone oil) was evenly coated on the inner wall of the mold (cylindrical, high-temperature custom molds with an inner diameter of 36 mm and height of 80 mm). The evenly mixed LCM slurry was poured into the mold, placed in a special steel container, and inserted into the high-temperature roller hearth furnace for heating and curing. After curing was complete, the mold was removed, and the ageing furnace was turned off. The cured samples were cooled and collected for analysis.

### 2.2. Physicochemical Properties of Resin Plugging System

#### 2.2.1. Microstructure

**Figure 9:**
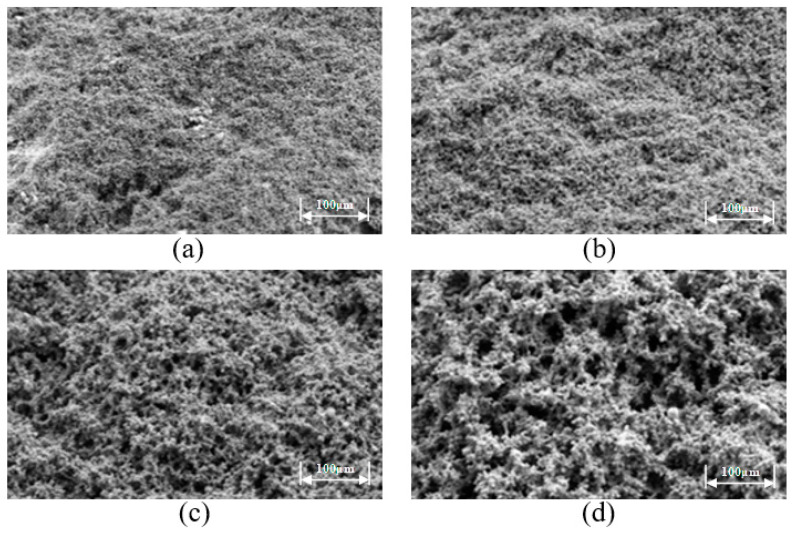
Microstructures of high-temperature-resistant, high-strength curable resin. (**a**) 1000× magnification, (**b**) 2000× magnification, (**c**) 5000× magnification, (**d**) 10,000× magnification.

The microstructures of the prepared high-temperature-resistant, high-strength curable resins were observed using SEM at magnifications of 1000×, 2000×, 5000×, and 10,000×, as shown in Figure 9. High-temperature-resistant, high-strength curable resins can form a chain or 3D mesh structure after curing and cross-linking. The resin crystal layers are tightly connected to each other, which to a certain extent can effectively increase the resilience and strength. The reason for this phenomenon is that a low-molecular-weight organosilicon compound was selected as the resin crosslinking agent, which contains epoxy, vinyl, amide, alkoxy and other reactive functional groups. One end can react with inorganic materials such as glass fibers, silicates, and metal oxides on the surface of the silyl alcohol group to generate covalent bonds. The other end can covalently bind to the resin, crosslinking the two incompatible materials to form a mesh structure. At the same time, during curing, the active groups contained in the resin, such as hydroxymethyl groups, amide bonds, and dimethylene ether bonds, will cross-link with formaldehyde, resulting in the formation of a 3D spatial network structure.

#### 2.2.2. IR Spectroscopic Analysis

**Figure 10:**
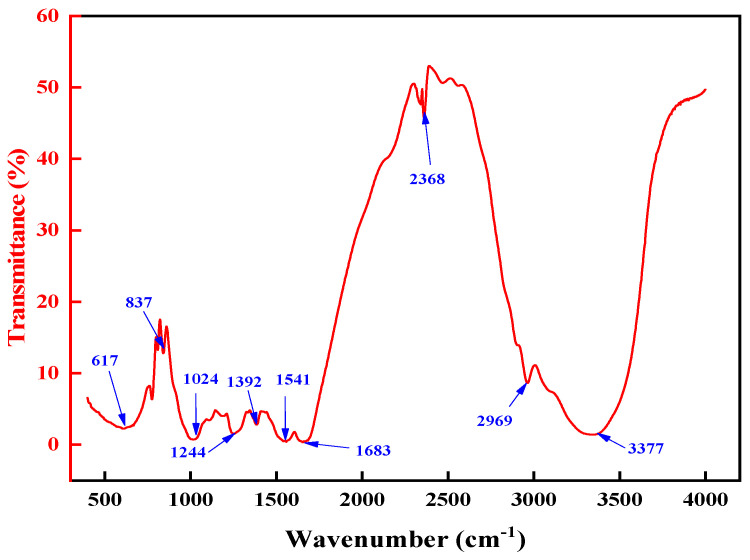
IR spectrum of resin plugging agent.

The IR spectra of the high-temperature-resistant, high-strength curable resin are shown in Figure 10. It can be seen that the characteristic peaks of resin are variable. More peaks corresponding to hydroxymethyl and ether bonds in the samples indicate that the target product is obtained. The reduction in –CH_2_OH, –NH–, and –NH_2_ content in the target product affirms that the reaction is complete. The telescopic vibration peaks for –NH–, –CH, and –C–O–C– appear at 3377 cm^−1^, 2969 cm^−1^, and 1024 cm^−1^, respectively. Their presence indicates that the high-temperature-resistant, high-strength curable resin was successfully prepared.

#### 2.2.3. Thermogravimetric Analysis

**Figure 11:**
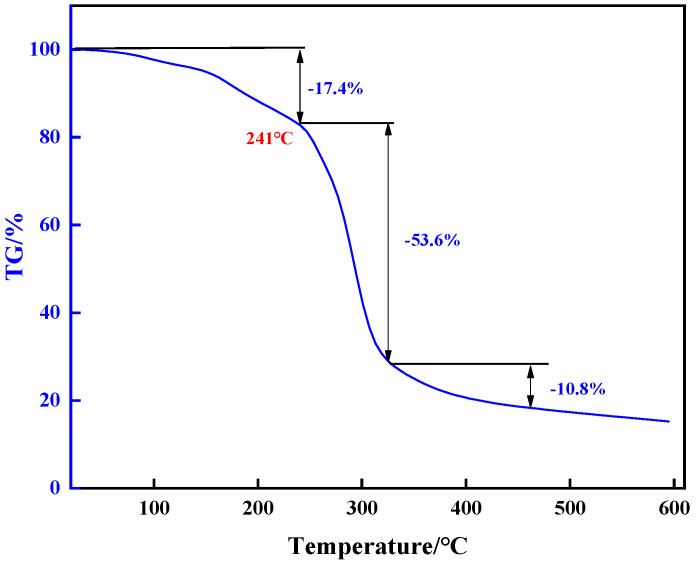
TGA profile of resin plugging agent.

The thermal stability of chemical bonds in the high-temperature-resistant, high-strength curable resin powders was examined using a thermogravimetric analyzer (TGA2, Geneva, Switzerland). First, the high temperature resistance and high strength curable resin was put into the oven at 105 °C to remove water and then ground into powder. For each measurement, 10–15 mg of high-temperature-resistant and high-strength curable resin samples were weighed at an initial temperature of 25 °C, a target temperature of 600 °C, and a heating rate of 20 °C/min. The experiment was carried out in a nitrogen atmosphere with a ventilation rate of 50 mL/min. The experimental results are shown in Figure 11.

As can be seen from the thermogravimetric curve in Figure 11, the initial decomposition temperature of the resin plugging system is about 241 °C, and the thermogravimetry is divided into three stages. The thermal decomposition of the chemical bond of the resin in the first stage occurs at about 50 °C, and the weight loss rate is 17.4% from 50 °C to 241 °C, mainly due to the presence of free water and bound water. The weight loss rate at 329.0 °C is about 53.6%, mainly due to the decomposition of amide groups. At 329.0 °C to 459.3 °C, the thermogravimetric rate reached 10.8%, mainly more hydroxymethyl, ether bond and other structural elements were formed, and the structural elements decreased after curing. The experimental results showed that the thermal decomposition of the resin plugging system is stable at about 300 °C, and the thermal stability of the chemical bond is good.

#### 2.2.4. Rheological Analysis

**Figure 12:**
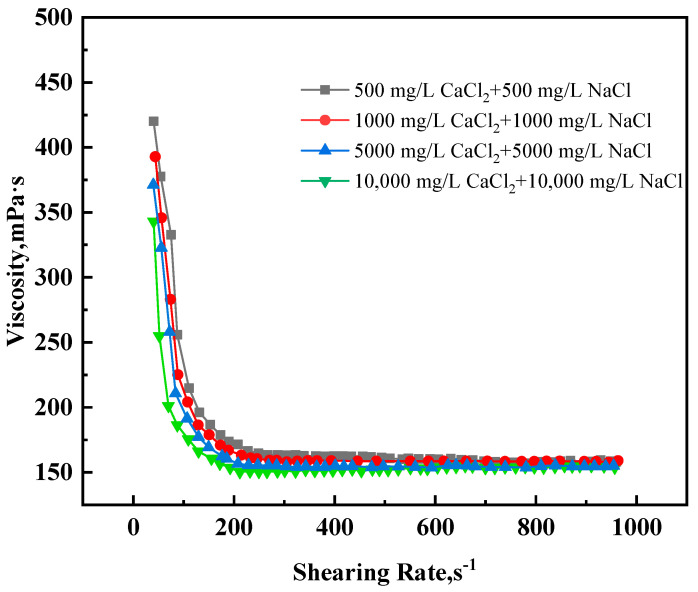
Variation in viscosity of resin plugging agent with shear rate.

The change in viscosity with shear rate before curing of the high-temperature-resistant, high-strength curable resin solution was tested using a HACKE rheometer. The test results are shown in Figure 12. The initial viscosity of the system lies between 300 and 400 mPa s, and the viscosity gradually decreases with the increase in shear rate, finally plateauing. The high-temperature-resistant and high-strength resin plugging agent exhibits shear thinning, which is favorable for on-site pumping, eliminating the need to use high-pressure pumping trucks.

The rheology of the high-temperature resistant, high-strength resin plugging agent at different mixing times under room-temperature conditions was studied, and the test results are shown in Table 9. The results show that the initial apparent viscosity of the resin system is 85 mPa s, 95 mPa s after mixing for 5 h, and 97.5 mPa s after mixing for 10 h. The resin system has a relatively stable apparent viscosity at room temperature, does not thicken, and will not solidify in the slurry tank.

The drilling fluid was mixed with the high-temperature-resistant, high-strength curable resin plugging agent in ratios of 3:7, 5:5, and 7:3 to study the effect of drilling fluid amount on the rheology of the system. The test results are shown in Table 10.

The addition of drilling fluid will have a certain effect on the rheological properties of the resin plugging agent. The apparent viscosity of the resin plugging system without adding drilling fluid is 85 mPa·s. After the drilling fluid was added in 3:7, 5:5, and 7:3 proportions, the apparent viscosity increased slightly due to hydration, and was 87.5 mPa·s, 97.5 mPa·s, and 97.5 mPa·s, respectively. Therefore, the slurry tank should be cleaned when the slurry tank is used in the field to prepare the resin plugging system.

### 2.3. Factors Affecting the Curing Effect of Resin Plugging System

#### 2.3.1. Effect of Temperature on Curing

**Figure 13:**
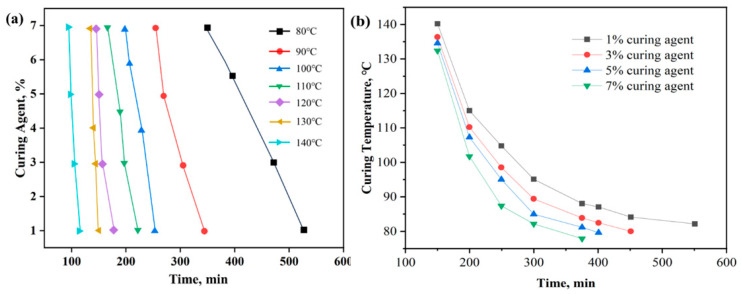
(**a**) Effect of curing agent concentration on curing time; (**b**) effect of curing temperature on curing time.

In the practical application of a resin plugging agent, different factors, particularly the curing time, must be considered. For the high-temperature-resistant, high-strength curable resin plugging agent, the curing time is affected by the temperature and the amount of curing agent added. When the curing agent concentration was 1% and the curing temperature was 80 °C, the curing time was 550 min; at 140 °C, the curing time was 151 min. When the curing agent concentration was 7%, the curing time was 300 min at 80 °C and 127 min at 140 °C. This indicates that the more curing agent added, the shorter is the curing time, and the higher the temperature, the faster is the curing process. When 1~7% of the curing agent is added at a temperature of 8~140 °C, the curing time is controllable from 120 min to 540 min. The results are shown in Figure 13.

**Figure 14:**
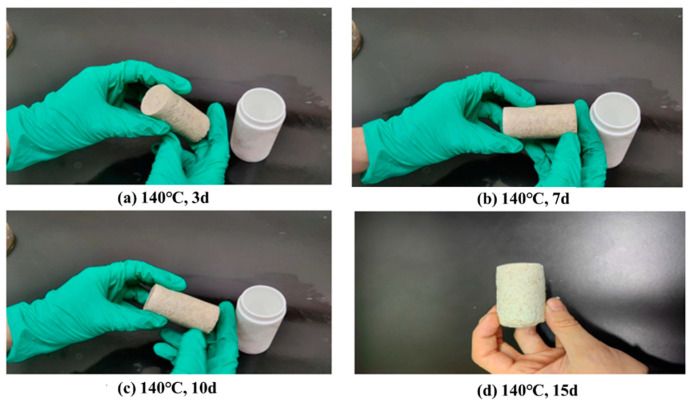
State of resin samples at varying ageing times.

**Figure 15:**
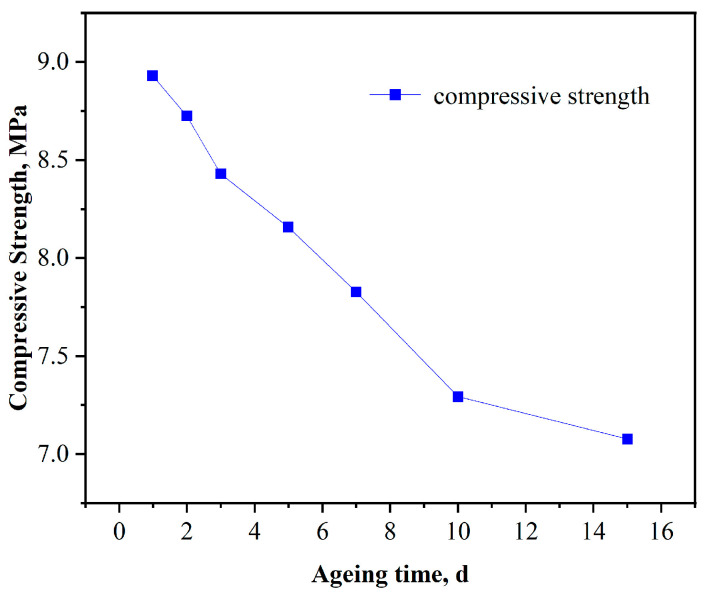
Effect of ageing time on curing strength.

The high-temperature-resistant, high-strength curable resin plugging agent was subjected to long-term high-temperature strength-loss experiments. At 140 °C, the resin plugging agent was aged for 1, 2, 3, 5, 7, 10, and 15 days (d). Some of the aged samples are shown in Figure 14. The compressive strength of the resin was tested, and the strength at 1 d of ageing was used as a comparative benchmark. The degree of strength reduction during long-term ageing and curing at high temperatures was studied, and the test results are shown in Figure 15.

The results of the study revealed that the compressive strength was 8.91 MPa at 1 d of ageing at 140 °C and 8.47 MPa after 2 d of ageing, with a decrease of 4.94% in cured strength; 8.35 MPa after 3 d, with a 6.29% decrease in cured strength; 8.21 MPa after 5 d, with a 7.86% decrease in cured strength; 7.81 MPa after 7 d, with a 12.35% decrease in cured strength; 7.25 MPa after 10 d, with a 18.63% decrease in cured strength; and 7.10 MPa after 15 d, with a 20.3% decrease in cured strength. According to these results, the high temperature stability performance is excellent.

#### 2.3.2. Effect of Mineralization on Curing Effectiveness

**Figure 16:**
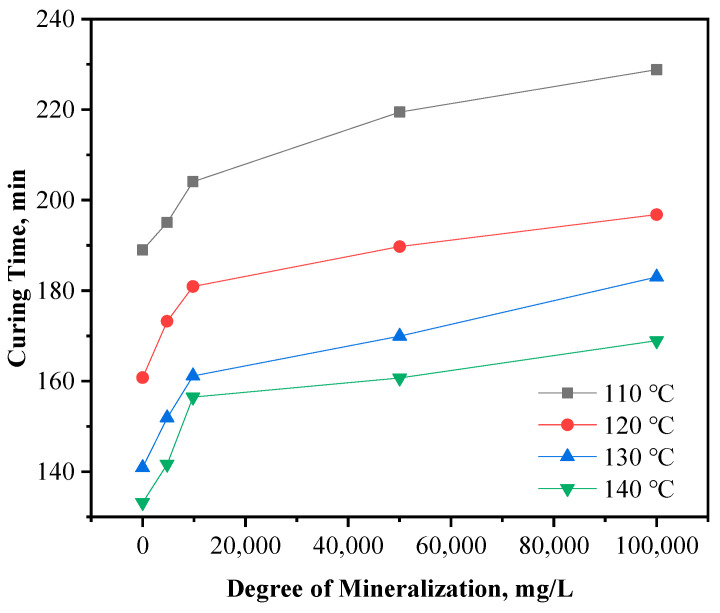
Effect of mineralization on curing time of resin plugging agent.

**Figure 17:**
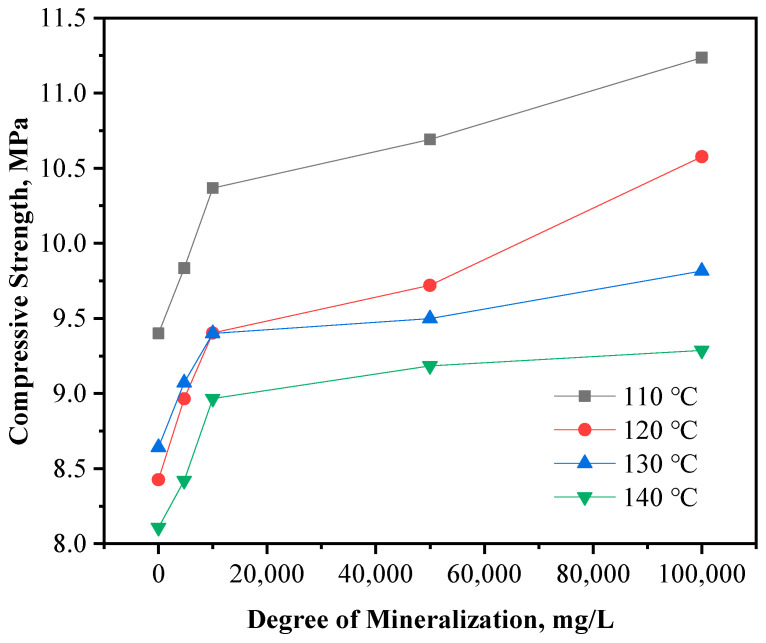
Effect of mineralization on cured strength of resin plugging agent.

Salt water with different salinity was used to simulate formation water with different salinity instead of deionized water to configure the resin plugging agent, and its curing time and curing strength were studied. The test results are shown in Figure 16 and Figure 17, respectively.

The results of the study show that curing of the resin plugging agent is effected as follows. In the case of water pollution in formations with varying mineralization degrees, the curing time was 189–229 min at 110 °C, and the strength was 9.3–11.0 MPa; 161–197 min at 120 °C, with a curing strength of 8.4–10.4 MPa; 141–183 min at 130 °C, with a strength of 8.6–9.7 MPa; and 133–169 min at 140 °C, with a strength of 8.1–9.2 MPa. These results indicate that the higher the mineralization degree, the longer is the curing time, whereas the higher the temperature, the shorter is the curing time.

#### 2.3.3. Effect of Drilling Fluid Contamination on Curing Effect

**Figure 18:**
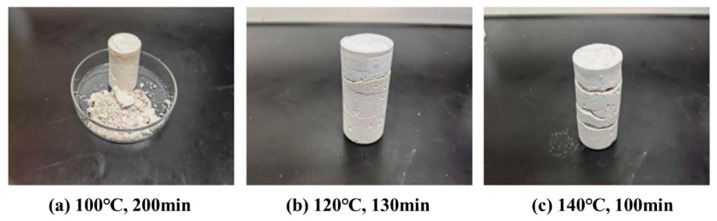
Curing effect of slurry of the resin plugging agent.

The curing effect of replacing water with drilling slurry in the prepared high-temperature-resistant, high-strength curable resin plugging agent was investigated. The agent was cured for 200, 130, and 100 min at 100, 120, and 140 °C, respectively. The curing results are shown in Figure 18, which reveal that the consolidated volumes are well formed with high strength at these three temperatures, but all samples were brittle, with varying degrees of cracking or fracture.

**Figure 19:**
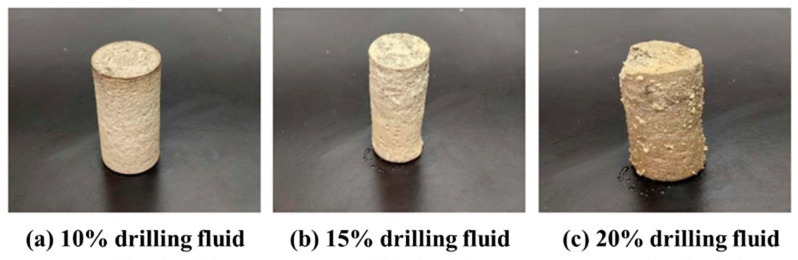
Curing effect of KCl polysulfone drilling fluid on resin system.

The resistance of the high-temperature-resistant, high-strength curable resin plugging agent to KCl polysulfone drilling fluid was investigated. The drilling fluid (10%, 15%, and 20%) was added to the resin plugging agent and cured in a heat roller for 120 min at 130 °C. The curing results are shown in Figure 19 and indicate that complete consolidation can be achieved, and the cured resin plugging agent has a certain degree of strength at all three levels of contamination; however, the overall strength gradually decreases with increasing level of contamination.

**Figure 20:**
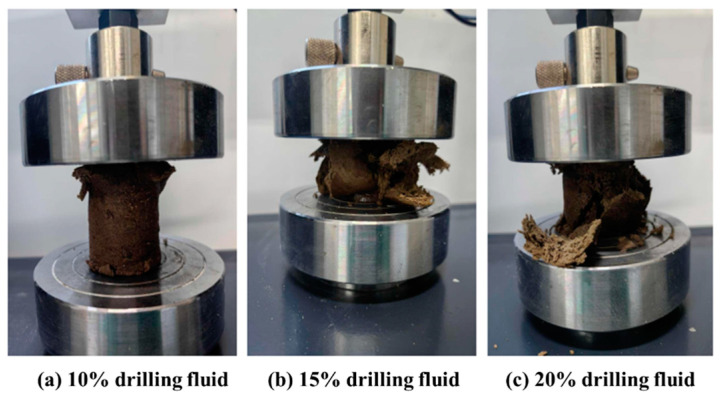
Compression test of samples formulated with varying concentrations of polysulfone drilling fluid.

Next, the compressive strengths of the formed solids were tested, as shown in Figure 20. The results are detailed in Table 11. The experimental results revealed that the compressive strength of the cementum was 6.44 MPa when 10% of the KCl polysulfone drilling fluid was added, 4.55 MPa when 15% of the KCl polysulfone drilling fluid system was added, and 4.55 MPa when 20% of the KCl polysulfone drilling fluid was added. The strength gradually decreased with increasing contamination.

From our comprehensive study, it is apparent that the high-temperature-resistant, high-strength resin plugging agent has a certain degree of resistance to contamination with drilling fluid. Relatively intact consolidated volumes can still be formed when the level of contamination is low, but the consolidation strength gradually decreases as the degree of contamination increases. Therefore, to avoid the residual drilling fluids in the slurry tank contaminating the resin plugging agent, cleanup work is needed before preparing the resin plugging agent and applying it in the field.

## 3. Conclusions

(1) In this study, a resin plugging system based on urea–formaldehyde resin was successfully developed for malignant well loss formation, which showed excellent performance under high temperature and pressure conditions.
(2) After curing, the compressive strength of the resin plugging material can reach 9.23 MPa. The material can achieve effective curing in the temperature range of 60 °C to 100 °C, the curing time decreases with the increase of temperature, and the curing time is 3.46 h at 140 °C.
(3) The resin plugging material forms a chain or three-dimensional network structure after curing, which effectively increases the toughness and strength of the material. FTIR and TG analysis further confirmed the thermal stability of the chemical bonds in the material. The initial decomposition temperature was about 241 °C, indicating that it had good thermal stability at about 300 °C.
(4) The effects of the curing temperature, the salinity, and drilling fluid pollution on the properties of resin plugging materials were studied. The results show that curing agent dosage and curing temperature are the key factors affecting the curing time, while salinity and drilling fluid pollution affect the curing strength and overall properties of the materials. After adding 20% KCl polysulfonate drilling fluid, the compressive strength of the consolidated body decreased to 4.55 MPa.

## 4. Materials and Methods

### 4.1. Materials

The reagents and materials used for the study are shown in Table 12.

### 4.2. Preparation of Resin Plugging System

A resin matrix, curing agent, and cross-linking agent were selected to form a base resin formulation with good curing strength. Then, the selected flow regulator and solid filler material were added to form a resin plugging material with good shear thixotropy and curing strength.

### 4.3. Rheology Testing

The rheological properties of the resin plugging agent affect the flow condition and injection of the plugging agent into the subsurface. In this experiment, the apparent viscosity of the resin plugging agent samples in the linear viscoelastic region was tested and evaluated using a HAAKE rheometer (Thermo Fisher Scientific, Waltham, MA, USA), in turn providing a measure of its pumpable performance. The rotor model used for the experiment was CC41/Ti (rotor diameter: 41 mm). The test sample temperatures were equilibrated for at least 30 min, and the temperature error was controlled to ±0.1 °C. The strain range was γ = 0.1–1000%, and the frequency range was 0–20 Hz. To ensure that the data were accurate, the rheology test was performed three times.

### 4.4. Mechanical Testing

The pressure capacity of consolidated lost circulation material (LCM) is an important criterion to judge the quality of the LCM. This experiment used an electronic universal testing machine (CMT4000, Shenzhen Xin Sansi Materials Testing Company, Shenzhen, China) to test the mechanical properties of compression at room temperature. Urea–formaldehyde resin LCM was cured at the bottom of a cylinder with a height and a diameter of 10 mm. The compression speed was set to 3 mm/min, and the stress–strain curve of the resin samples under compression was recorded.

### 4.5. Infrared Spectroscopic Analysis

In this experiment, an infrared (IR) spectrometer was used to scan the cured samples as a basis for judging the extent to which the target curing reaction had occurred. First, the IR spectrometer was warmed up for at least 15 min, the parameters were set, and then the spectral range and optical range of the instrument were set according to the experimental requirements. Then, the sample to be tested and potassium bromide were ground together according to a mass ratio of 1:100 (sample:KBr), and the powder was pressed into a pellet using a hand-held die press under a pressure of 15–20 MPa. Then, the scanning mode was selected, and the scanning parameters were set to a range of 1000–3500 cm^−1^ and interval of 2 cm^−1^.

### 4.6. Thermogravimetric Analysis (TGA)

In this experiment, a thermogravimetric analyzer was used to analyze the thermal stability of the cured resin plugging agent and also to analyze the curing. At the beginning of the experiment, the balance protective gas was turned on, allowing nitrogen to be added at a rate of 20 mL/min. After completing the instrument self-construction, the dried and crushed resin sample powder was placed into a special crucible on the sensor, and the sample was heated to the target temperature at a rate of 15–25 °C/min. The mass loss was recorded after a period of time.

### 4.7. Microstructural Analysis

The microstructure of the cured resin plugging agent can directly reflect the quality of the curing condition. First, the sample was processed, and the sample to be measured was cut and processed to obtain the measurement cross-section. Owing to the poor conductivity of the sample, it was necessary to sputter it with gold. Then, the microstructure of the resin plugging agent sample was characterized using a field-emission scanning electron microscope (FE-SEM; S-4700, Hitachi, Tokyo, Japan). Imaging was performed at 10 kV.

## Figures and Tables

**Table 1:** Curing of different resin matrices.

Curing Effect	40 °C	60 °C	80 °C	100 °C
Urea–formaldehyde resin	Incurable	1 h, moderate strength	2 h, high strength	3.46 h, high strength
Phenolic resin	Incurable	1.5 h, low strength	2.5 h, moderate strength	4 h, moderate strength
Epoxy resin	Incurable	0.5 h, relatively low strength	1 h, low strength	2.3 h, low strength
PVA	Rapid curing, low strength	Rapid curing, low strength	Rapid curing, but almost no strength	Watery, translucent liquid
Melamine resin	Incurable	Milky white water with precipitation	Milky white water with sediment and floaters	Milky white watery with some clumping

**Table 2:** Settling stability results of five resins.

Curing Effect	1 h	3 h	8 h	24 h
Urea–formaldehyde resin	Uniformly distributed	Uniformly distributed	Uniformly distributed	Uniformly distributed
Phenolic resin	Uniformly distributed	Uniformly distributed	Overall homogeneous, with small amount of sedimentation	Overall homogeneous, with small amount of sedimentation
Epoxy resin	Uniformly distributed	Uniformly distributed	Uniformly distributed	Uniformly distributed
PVA resin	Overall homogeneous, with partial precipitates	Beginning to stratify, with white precipitates	Significant stratification with relatively abundant sedimentation	Complete stratification
Melamine resin	Rapidly cured to a solid	Solid	Solid	Solid

**Table 3:** Summary of selected resin types.

Resin Type	Curing Temperature	Curing Condition
Epoxy resin	100 °C	Unmolded, viscoelastic, resilient
	120 °C	Unmolded, viscoelastic, resilient
	140 °C	Unmolded, viscoelastic, resilient
Phenolic resin	100 °C	Unmolded, viscoelastic, resilient
	120 °C	Unmolded, viscoelastic, resilient
	140 °C	Unmolded, viscoelastic, resilient
Urea–formaldehyde resin	100 °C	Good forming/good strength
	120 °C	Good forming/better strength
	140 °C	Good forming/better strength

**Table 4:** Summary of selected resin concentrations.

Resin Concentration	Curing Condition
12%	Cured without forming, unable to test strength
15%	Curing with forming, tensile strength of 7.51 MPa
20%	Curing with forming, tensile strength of 9.23 MPa
25%	Curing with forming, tensile strength of 9.41 MPa

**Table 5:** Curing agent plan.

Plan No.	Curing Agent Type
Plan 1	No curing agent
Plan 2	Ammonium curing agent
Plan 3	Ammonium composite curing agent
Plan 4	Latent curing agent

**Table 6:** Maximum consolidation strength for each plan.

Curing Agent Plan	Curing Temperature/°C	Curing Time/min	Curing Strength/MPa
Ammonium curing agent	120	130	4.32
Ammonium composite curing agent	120	130	5.21
Latent curing agent	120	130	7.13

**Table 7:** Summary of selected crosslinker concentrations.

Plan No.	Crosslinking Agent Selection	Curing Condition	Curing Strength/MPa
Plan 1	0.5% hydroxyethyl methacrylate	Inability to completely consolidate	–
Plan 2	0.2% divinylbenzene	Consolidation, but with low strength and some breakage	5.15
Plan 3	0.5% hydroxyethyl methacrylate + 0.2% divinylbenzene	Able to form a complete solid with high strength	7.07

**Table 8:** Filling materials with varying densities used in this study.

Filler Material	Density/(g/cm^3^)	Main Characteristics
Barite	2.5–4.2	Chemically stable and can be used as a heavy weight additive
Nano-SiO_2_	2.3–2.6	Improves ageing, chemical, and strength properties of other materials
Quartz sand	2–2.65	Hard, wear-resistant, and chemically stable
Fiber	1.5–1.52	The tensile strength reaches 500 MPa, which can increase structural integrity and impart strong resistance to acid and alkali
Rubber granule	0.96–1.02	Anti-ageing effect, long life, and easy maintenance
Walnut shell	0.70–0.75	High stress resistance and chemical stability

**Table 9:** Rheology of resin system after mixing for varying durations at room temperature.

Rheology	_Φ600_	_Φ300_	_Φ200_	_Φ100_	_Φ6_	_Φ3_	First Cut/Final Cut	Apparent Viscosity AV	Plastic Viscosity PV	Dynamic Shear Force γP
Initial state	170	117	88	57	11	8	4.5/9	85	53	32
Mixing 5 h	190	175	145	105	25	18	8.5/10	95	15	80
Stirring 10 h	195	183	149	105	26	18	8.5/9.5	97.5	12	85.5

**Table 10:** Effect of drilling fluid amount on the rheology of resin systems.

Rheology	_Φ600_	_Φ300_	_Φ200_	_Φ100_	_Φ6_	_Φ3_	First Cut/Final Cut	Apparent Viscosity AV	Plastic Viscosity PV	Dynamic Shear Force YP
Well slurry	15	11	9	6	3	3	5/6	7.5	4	3.5
resin	170	117	88	57	11	8	4.5/9	85	53	32
Well slurry: resin 3:7	175	155	114	71	18	9	5/7	87.5	20	67.5
Well slurry: resin 5:5	195	145	110	66	15	12	7/10	97.5	50	47.5
Well slurry: resin 7:3	195	178	130	68	22	21	9/11	97.5	17	80.5

**Table 11:** Compressive strength of KCl polysulfone drilling fluid on resin system.

Samples	Bottom Diameter/mm	Maximum Force/N	Compressive Strength/MPa
10% KCl drilling fluid contamination	35.23	6276	6.44
15% KCl drilling fluid contamination	35.95	5463	5.38
20% KCl drilling fluid contamination	34.97	4367	4.55

**Table 12:** Experimental materials.

Reagent	Purity	Manufacturer
Urea–formaldehyde resin	99.0%	Shanghai Aladdin Bio-Chem Technology Co., Ltd. (Shanghai, China)
Phenolic resin	98.0%	Shanghai McLean Biochemical Technology Co., Ltd. (Shanghai, China)
Epoxy resin	99.0%	Saan Chemical Technology (Shanghai, China) Co., Ltd.
Betaine monohydrate	99.5%	Shanghai McLean Biochemical Technology Co., Ltd.
Silane coupling agent, KH-570	99.0%	Changzhou Runxiang Chemical Co., Ltd. (Changzhou, China)
Ammonium chloride	99.0%	Saan Chemical Technology (Shanghai) Co., Ltd.
Hexamethylenetetramine	98.0%	Saan Chemical Technology (Shanghai) Co., Ltd.
Sodium carboxymethylcellulose	96.0%	Sinopharm Chemical Reagent Co., Ltd. (Shanghai, China)
Barite	99.7%	Sinopharm Chemical Reagent Co., Ltd.
Deionized water		In-house made

## Data Availability

The original contributions presented in the study are included in the article, further inquiries can be directed to the corresponding author/s.
